# Exploiting indigenous knowledge of subsistence farmers’ for the management and conservation of Enset (*Ensete ventricosum* (Welw.) Cheesman) (*musaceae* family) diversity on-farm

**DOI:** 10.1186/s13002-016-0109-8

**Published:** 2016-09-01

**Authors:** Zerihun Yemataw, Kassahun Tesfaye, Awole Zeberga, Guy Blomme

**Affiliations:** 1 Areka Agricultural Research Center, P.O.Box 79, Areka, Ethiopia; 2 Department of Microbial, Cellular and Molecular Biology, Addis Ababa University, Addis Ababa, Ethiopia; 3 Addis Ababa University, Institute of Biotechnology, P.O. Box 32853, Addis Ababa, Ethiopia; 4 Ethiopian Biotechnology Institute, Ministry of Science and Technology, P.O. Box 32853, Addis Ababa, Ethiopia; 5 Southern Agricultural Research Institute, P.O.Box 06, Hawassa, Ethiopia; 6 Bioversity International, Ethiopia Office, P. O. Box 5689, Addis Ababa, Ethiopia

**Keywords:** Enset, Ethiopia, Indigenous knowledge, Landraces, On-farm diversity, On-farm management

## Abstract

**Background:**

Enset (*Ensete ventricosum* (Welw.) Cheesman) belongs to the order *sctaminae*, the family *musaceae*. The *Musaceae* family is subdivided into the genera *Musa* and *Ensete*. Enset is an important staple crop for about 20 million people in the country. Recent publications on enset ethnobotany are insignificant when compared to the diverse ethnolingustic communities in the country. Hence, this paper try to identify and document wealth of indigenous knowledge associated with the distribution, diversity, and management of enset in the country.

**Methods:**

The study was conducted in eight ethnic groups in the Southern Nations, Nationalities and Peoples’ Regional State. In order to identify and document wealth of indigenous knowledge, the data was collected mainly through individual interviews and direct on-farm participatory monitoring and observation with 320 farm households, key informant interviews. Relevant secondary data, literature and inter-personal data were collected from unpublished progress report from National Enset Research Project, elderly people and senior experts.

**Results:**

Enset-based farming system is one of a major agricultural system in Ethiopia that serves as a backbone for at least ¼ of country’s population. Farmers used three morphological characters, two growth attributes, disease resistance and five use values traits in folk classification and characterization of enset. A total of 312 folk landraces have been identified. The number of landraces cultivated on individual farms ranged from one to twenty eight (mean of 8.08 ± 0.93). All ethnic groups in the study area use five use categories in order of importance: *kocho* yield and quality, *bulla* quality, *amicho* use, fiber quality and medicinal/ritual value. Of the 312 landraces 245 landraces having more than two use types. Management and maintenance of on-farm enset diversity is influenced by systematic propagation of the landraces, exchange of planting material and selective pressure.

**Conclusion:**

It can be concluded that the existing farmers’ knowledge on naming, classification and diversity should be complemented with maintenance of the creative dynamics of traditional knowledge and transmission of the knowledge are crucial for constructing sustainable management.

## Background

The Ethiopian highlands are a center of genetic diversity for enset, tef, sorghum, barley and finger millet [1]. Enset (*Ensete ventricosum* (Welw.) Cheesman) belongs to the order *sctaminae*, the family *musaceae*. The *Musaceae* family is subdivided into the genera *Musa* and *Ensete* [2]. Enset is an important staple crop for about 1/4 (20 million) of the population of the people living in the densely populated regions of South and Southwestern Ethiopia. The crop is grown in mixed subsistence farming systems, often in association with coffee, multi-purpose trees, and annual food and fodder crops [3]. Enset is also used for livestock feed, fuel wood, construction materials, containers, and as a provider of shade to intercropped annual or perennial crops [4]. It is cultivated between 1500 and 3100 m above sea level (m.a.sl), where daily average minimum and maximum temperatures are 8 and 27 °C, respectively [5].

The major food types obtained from enset are *kocho*, *bulla* and *amicho. Kocho* is fermented starch obtained from decorticated (scraped) leaf sheaths and grated corms. *Bulla* is obtained by squeezing out the liquid containing starch from scraped leaf sheathes and grated corm and allowing the resultant starch to concentrate into white powder. *Amicho* is boiled enset corm pieces, mainly obtained from young enset plants that are prepared and consumed in a similar manner to other root and tuber crops [6].

Studies indicate that numerous enset cultivars were identified in each region and the observed genetic diversity in cultivated enset in a particular area appears to be related to the extent of enset cultivation and the culture and distribution pattern of the different ethnic groups [7].

A clear understanding of the diversity and distribution of enset is important for crop improvement programs and for managing genetic resources. To measure the status of crop diversity in the field the most common method is counting named varieties. There are two main landrace diversity indices, namely: cultivar richness, which represents the number of landraces in a community, and cultivar evenness, representing the relative abundance of the individuals among the various landraces present in the community [8, 9]. For farmers, genetic diversity means varietal diversity, which farmers can clearly distinguish on the basis of agro-morphological traits, phenological attributes, post-harvest characteristics, and differential adaptive performance under abiotic and biotic stresses [10].

Indigenous technical knowledge is the tool by which local people interact with the environment in order to meet needs and goals ranging from survival goals to that of achievement and esteem [11]. It is knowledge, which is unique to a local area, culture, or society, passed down from one generation to the next, usually through oral tradition. Indigenous knowledge has to do with theories, beliefs, practices, and technologies that local people have elaborated without any assistance from the modern, formal and scientific communities and/or institutions [12]. Indigenous people have a long tradition in maintaining biodiversity as a sustainable resource. Farmers have played and still continue to play a tremendous role in developing and nurturing crop genetic diversity. Many studies have shown that farmers in developing countries have intimate knowledge of environmental processes and make rational resource management decisions based on that knowledge [13].

The southern and southwestern part of Ethiopia has an extraordinary biological and cultural diversity. Recent publication on enset ethnobotany including those by [13, 14] attempt to document farmers’ indigenous knowledge on enset in some cultural groups at specific location. However, those documentations are insignificant when compared to the diverse ethnolingustic communities in the country. This paper seeks to contribute towards filling this knowledge gap, based on an empirical study of enset farmers in Ethiopia. The paper address the following main question: what are farmers’ knowledge associated with the distribution, diversity, and management of enset in the country? The underlying assumption behind this question is that all farmers are equally likely to be knowledgeable about the crop.

Hence, the objectives of this study was to identify and document wealth of indigenous knowledge for folk naming, classification, distribution and abundance of enset landraces and understanding the corresponding knowledge related to utilization, management and conservation of enset landraces.

## Methods

### The study area

**Fig. 1:**
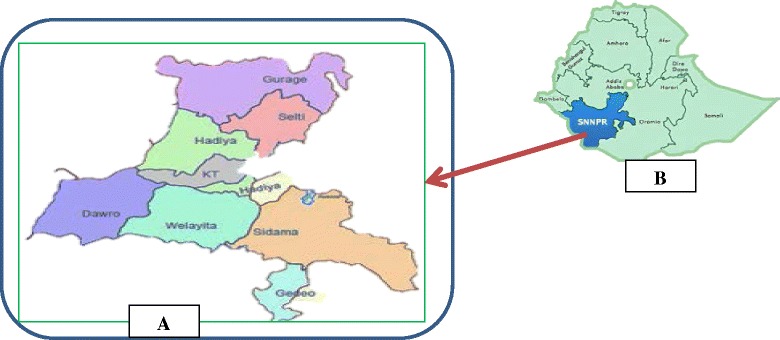
**a** Detail zones map of the study region. **b** Map and Position of the study region in Ethiopia

The SNNPR is one of the regions in Ethiopia. It is located in south and southwestern part Ethiopia, 4.43°–8. 58° N latitude and 34.88°–3914° E bordering Kenya to the south and South Sudan to the west and southwest, the Ethiopian region of Gambela to the northwest, and the Ethiopian region of Oromia to the north and east (Fig. 1). The region has a total area of 110,931.9 square kilometers lying within elevations of 378 to 4207 m above sea level [15]. The annual temperature is less than 10 °C in the extreme highlands to over 27 °C in the lowlands of the south. The regions are sub divided in to zones, which are organized in to weredas/districts. The zones are named based on the name of the dominant ethnic group for that specific location. The Regions are sub-divided into Zones, which are organized into weredas/districts. Within weredas, kebeles are the smallest administrative units.

### Study site selections

The study was conducted in eight ethnic groups/zones (Dawro, Gedeo, Gurage, Hadiya, Kembata-Tembaro, Sidama, Silte, and Wolaita) in the Southern Nations, Nationalities and Peoples’ Regional State (SNNPRS). The eight ethnic groups were selected for the following reasons:

1. The crop has coexisted with the people for centuries and enset production is pre-dominantly based on farmers’ varieties. Hence, farmers’ expected to have an established folk naming, classification system of appraisal of enset.
2. The ethnic groups had rich on-farm genetic resources of enset that made it suitable to study ethnobotanical descriptions [13, 14] of enset.
3. In the region, enset cultivation is the center of the cropping system in which the entire farming system is based and the crop is the major food security and livelihood source [13, 14].

Two wereda were selected from each ethnic groups based on enset diversity (Table 1). Then, two kebeles which are major enset growing areas were purposively selected from each wereda/district based on the importance of enset cultivation and information about enset distribution obtained from the Departments of Agriculture and Natural Resource of the respective zones.

**Table 1:** Description of surveyed woredas and their agro-ecological characterization

No.	Zone	Woreda	Elevation(m.a.sl)	Minimum and Maximum T^o^	Annual RF (mm)
1	Gedeo	Bulle	2428	15–22.5	1200–1800
		Gedebe	2171	12–21	800–1150
2	Wolayta	Boloso Sore	1871	14–25	1100–1500
		Sodo Zuria	2200	14–25	1100–1800
3	Guragie	Cheha	2638	11–21	1100–1850
		Geta	2731	10–22	1000–1800
4	Kembata-Tembaro	Angacha	2465	15–24	900–1750
		Doyogena	2748	10–22	1000–1800
5	Silte	Mirab Azerenet	3191	11–18	950–1900
		Alicho Werero	2707	12–22	700–2000
6	Hadiya	Dunna	2619	11–21	1100–1850
		Misha	2367	12–21	800–1150
7	Daworo	Mareka	2482	12–21	1200–1800
		Tocha	2754	12–21	1200–1800
8	Sidama	Dalle	1855	12–26	1000–1800
		Hulla	2759	10–17	900–1850

### Sampling

Multistage sampling technique was employed for selection of samples, zones, weredas and kebeles. All stages were selected purposefully from high (>2500 m.a.sl) and mid altitude (1500–2500 m.a.sl) [16] areas in consultation with stakeholders engaged in the subsector. Eight Zones, two weredas from each zone (16 wereda) and two Kebele Administration (KA) (Kebeles are the lowest administrative unit) from each wereda (32 KAs), were selected purposefully based on agro-ecology variant. A total of 320 households (40 household heads from each ethnic) over the selected ethnic groups in the two crop ecologies were directly monitored on farms. The survey focused on the investigation of farmers’ folk knowledge for naming, classification, diversity and management of enset landraces in the region.

### Data collection

Diverse data collection methods were employed in order to understand the many features for the acquirement of local knowledge of enset naming, classification, diversity and management in the center of diversity. The data collection was conducted mainly through: i) individual interviews and direct on-farm participatory monitoring and observation, ii) key informant and focus group discussions, and iii) secondary data and literature survey.

### Individual interviews and direct on-farm participatory monitoring and observation

Before interviews were performed, informal conversation was conducted with 20 inhabitants of the enset community with the objective of determining which type of information needed to be collected. Based on these conversations, semi-structured interviews were designed and data collected with the head of the household or the person responsible for maintenance of the enset plantation. Three hundred twenty farmers were interviewed and directly monitored on farms, over the selected weredas in order to assess the farmers’ ethnobotany knowledge on enset.

The questionnaire covered different topics such as information about the study area, landholdings, crops commonly grown and specific information on the use and management of enset. The detailed information was focused on enset diversity, cultural practices, source of planting materials, and traditional use values of enset. The respondents were also asked about their perception on enset production constraints and their indigenous knowledge about the disease.

### Key informant interviews

In order to assess the general indigenous knowledge of farmers’ in each ethnic group: key informants up to five per KA, community leaders, local administrations, and MOA (Ministry of Agriculture), and other members in each ethnic site were interviewed.

### Secondary data and literature survey

National Enset Research Project progress report was visited for secondary data and personal communication and discussion with elderly people and senior experts in line with ethnobotany tradition of enset. Literatures on enset culture were reviewed from published and unpublished sources and reports.

### Data analysis

Informal discussion with elderly farmers, and key informants were carried out to validate the information gathered from individual interviews. Lists of all landraces described throughout the study area were summarized after grouping known synonyms or names that refer to the same landraces in each wereda with the help of elderly farmers.

Collected survey data were subjected to descriptive statistics (frequencies, percentages, and average) using SPSS Ver. 16. Landrace richness, diversity and dominance per farm were calculated using Microsoft excel 2010. Richness was calculated as the total number of landraces per farm and averaged this figure per ethnic group. Abundance was calculated as the total number of individual plants of each landraces per farm/household. Frequency was estimated as the number of individuals of a landraces with respect to the total number of landraces composing the enset farm. With these parameters we calculated the ecological importance index of each cultivar per farm.

The Shannon and Weaver [17] and Simpson [18] diversity indices are two of the most widely used measures of heterogeneity [19]. Both of them were calculated for all the surveyed zones. The Shannon–Weaver diversity index accounts for both abundance and evenness of the landraces present and can be increased either by greater evenness or more unique landraces. It was calculated using the formula, *H' = − Σ pi ln pi*, [19]. Where pi, the proportional abundance of the i^th^ landrace. Then we calculated the dominance as a measure representativeness of each landrace through the Simpson index. Simpson’s Index of Diversity (1 – D) was computed for all the zones and all the landraces using the function: Simpson’s Index of Diversity (1-D) = 1-∑ (n/N)^2^.

$D={\displaystyle \sum_{i-1}^n\frac{\Big(ni\kern0.5em \left(ni-1\right)}{\Big(N\left(N-1\right)}}$ where, n_i_ = the frequency of the i^th^ landrace, frequency being the number of farms in which the landrace is found in the district, and *N* = the total number of farms surveyed in the zone.

Equity, the proportion of the observed diversity with respect the maximum diversity expected was calculated through the Pielon index: J = H’/H’max, in which J is equity; H’ = diversity; H’max = maximum diversity, H’max was calculated as the ln(S) S being the number of landraces in a sample. Pearson’s correlation coefficient was used to compare diversity and distribution values at different ethnic groups.

We used a multiple use curve [20] concept to describe the rate at which ethnobotanical data is collected, check whether the essential part of the available information on the landraces had been collected. This curve plotted the cumulative number of uses recorded against the number of informants. To analyze the use values of the landraces, we regrouped the uses into broad categories, where each category contained uses of a similar nature. In this way, three main categories were created, namely; food (*kocho* yield and quality, *bulla* quality, *amicho* use), fiber (fiber quality) and medicinal/ritual categories. Food and medicinal categories refer to use by both humans and animals.

## Result

### Strategic importance of enset

**Fig. 2:**
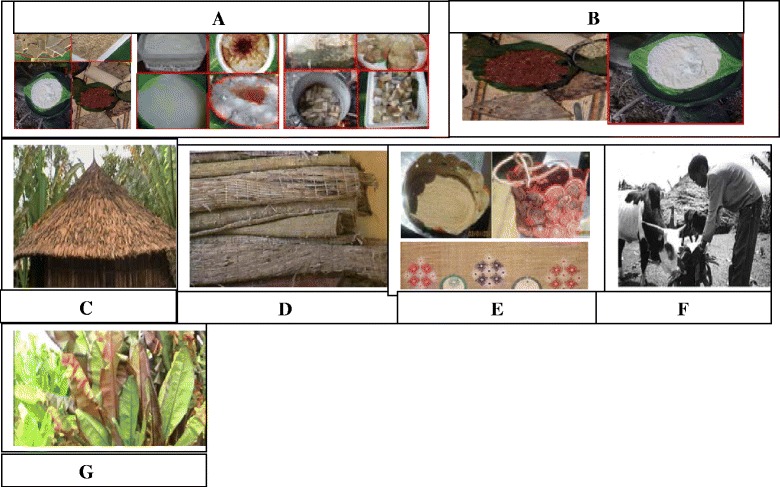
Different uses of enset. **a** food products (*Koch*, *bulla and Amicho*). **b** Used as plate. **c** Enset leaf sheath thatching of huts. **d** used as a bed (**e**) used as bag and decoration. **f** A young boy feeding a cow with enset leaf. **g** enset landraces used for medicinal use value

Enset-based farming system is one of a major agricultural system in Ethiopia that serves as a backbone for at least one-fifth of country’s population. Enset has been selected as a typical multipurpose crop of which every part is thoroughly used for food, feed, medicinal, construction and ornamental purposes. Throughout the growth stage the corm, pseudostem and leaves are sued for various purposes. Enset is intimately associated with the daily lives of the farmers. Owing to these facts, farmers indicated that, ‘enset is everything for us’. ‘It is our food’ (Fig. 2a), ‘it is our plate’ (Fig. 2b), ‘it is our house’ (Fig. 2c), ‘it is our bed’ (Fig. 2d), ‘it is our bag’ (Fig. 2e) ‘it is our cattle feed’ (Fig. 2f) and it is our medicine (Fig. 2g). It is the most important crop in the farmers’ livelihoods and security.

### Indigenous knowledge in naming and classification

**Fig. 3:**
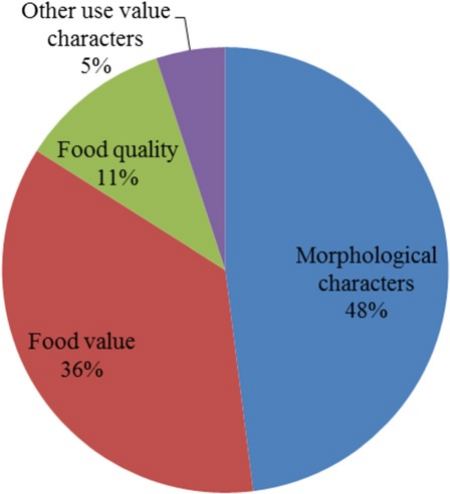
Proportional importance of different selection criteria’s in all the communities studied in the SNNPRS, Ethiopia

*Ensete* is the genus name, while different ethnic group use different vernacular terms as a local name for *Ensete*. In the study area, *Ensete ventricosum* is identified through various local names (Table 2). Farmers in the study area use a combination of similar criteria to name and classify enset landraces (Table 3). They classify their landraces and give different names based on several attributes that distinguish these landraces from one another. Three morphological characters (midrib color, petiole color, and leaf color), Growth attributes (vigor, maturity), disease resistance and use value food (*kocho* yield and quality, *bulla* quality, *amicho* use), fiber quality and medicinal value were the major criteria used by farmers. The interviewees referred first to the morphological characters (48 %) (Fig. 3) of any enset landrace when asked for key classifying characteristics. The food usage, food quality, and other use value characters were usually mentioned as those of second importance for classification. It is witnessed that the names given by all enset growing farmers to the different landraces and the classification criteria are generally consistent.

**Table 2:** Local names of *Ensete ventricosum*

Ethnic group	Local name
Dawro	U’tt’a
Gedeo	Workicha
Gurage	Aset
Hadiya	Weisa
Kembata-Tembaro	Wessa
Sidama	Wessie
Silte	Weisa
Wolaita	Utta

**Table 3:** Farmers’ criteria for classification of enset clones in, the eight Ethnic groups and frequency distribution of the 320 respondents

Trait	Descriptor state	Respondents
Plant vigor	Poor (<4 m)	22
	Medium (4–6 m)	40
	High (>6 m)	38
Maturity (cycle duration)	Early (<4 years)	33
	Intermediate (4–5 years)	43
	Late (>6 years)	24
*Kocho* yield	Low (<9.9 t ha^−1^ yr^−1^)	9
	Medium (9.9 to 20 t ha^−1^ yr^−1^)	53
	High (>20 t ha^−1^ yr^−1^)	38
*Bulla* quality	Not good	12
	Good	88
Corm use	Not used	58
	Used	42
Fiber quality	Low	23
	Medium	51
	High	26
Medicinal value	Not used	88
	Used	12
Disease response	Susceptible	80
	Intermediate	8
	Tolerant	12
Petiole color	Green	45
	Green yellow	1
	Pink purple	4
	Red	29
	Red purple	11
	Purple	5
	Brown	4
	Black	1
Midrib color	Green	36
	Green yellow	1
	Red	17
	Red purple	16
	Pink	14
	Pink purple	10
	Purple brown	4
	Black	1
	Ivory	1
Leaf color (upper surface)	Light green	61
	Medium green	24
	Green	15

### Level of on-farm richness, diversity and pattern of use

We recorded a total of 440 folk varieties (landraces) across the eight ethnic groups. From the total 128 (29 %) landraces shared the same name in at least two ethnics and the total number of landraces reduced to 312 (Table 14). As farmers over years have selected their landraces for multipurpose values, they do group them according to the use values landraces renders. Each landrace is clearly distinguished by its vernacular name and peculiar characteristics. Of the 312 landraces 288 were reported to be known by all of the interviewees, whereas the 24 landraces were found in less than 5 % of the respondents’ farm.

Based on the total number of different landraces recorded (richness of the ethnic group) and the number of enset landraces per farm, Dawro farmers’ had the highest number of landraces (75) accounting for 24 % of the total number of recorded landraces across the study area. In contrast, the lowest richness was found in Gedeo farmers’ with 26 landraces accounting for 8.33 % of the total number of recorded landraces (Table 4). The number of landraces cultivated on individual farms ranged from one to twenty eight (mean of 8.08 ± 0.93) (Table 4). Average number of landraces per farm ranged between 10.43 for Silte to 3.55 for Wolaita. Dawro and Sidama with 10.2 and Gurage with 9.45 landraces per farm had high farm level richness (Table 4).

**Table 4:** Enset clone diversity in the eight ethnic groups, Southern Ethiopia, Expressed as richness, Simpson(1-D) and Shannon (H') diversity indices, and Evenness

Districts	Richness (%)	Mean richness / farm	Minimum richness	Maximum richness	No. of unique landraces	1-D	H'	Evenness
Dawro	75 (17.04)	10.2	1	28	21	0.97	3.71	0.86
Gedeo	26 (5.91)	4.75	1	8	20	0.9	2.6	0.8
Gurage	63 (14.32)	9.45	3	21	15	0.96	3.69	0.89
Hadiya	51 (11.59)	8.19	4	15	20	0.95	3.4	0.86
Kembata-Tembaro	66 (15)	7.83	3	15	15	0.96	3.62	0.86
Sidama	62 (14.1)	10.27	3	28	45	0.96	3.5	0.85
Silte	69 (15.68)	10.43	3	24	20	0.96	3.67	0.87
Wolaita	28 (6.36)	3.55	2	7	15	0.93	2.86	0.86

Diversity indices for the eight ethnic groups studied were computed from the numbers of landraces present on the 40 farms within the ethnic (Table 4). Although ethnics differed in richness, they were similar in diversity. The Simpson’s 1-D ranged between 0.97 (Dawro) to 0.9 (Gedeo), H′ ranged between 3.71 for Dawro to 2.6 for Gedeo, while evenness also had a very narrow range: 0.89 for Gurage to 0.8 for Gedeo (Table 4). Both the H’ and 1-D indices were highly correlated with landrace number at each ethnic (*r* = 0.90 and 0.70). All these values indicate the high enset diversity in these eight ethnic groups.

**Fig. 4:**
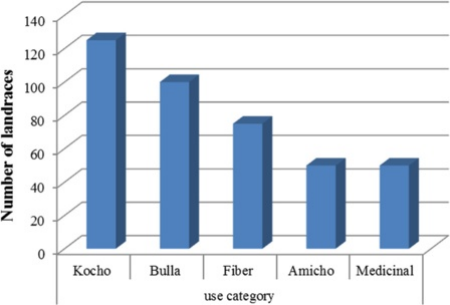
Uses of the landraces recorded in the home gardens of all the communities studied in the SNNPRS, Ethiopia

**Fig. 5:**
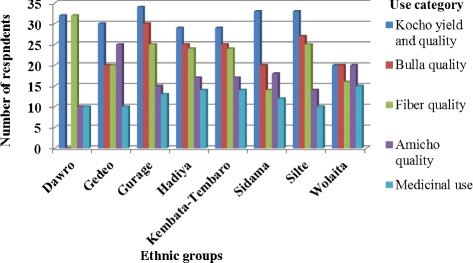
Comparative analysis of use category in each ethnic group studied in the SNNPRS, Ethiopia

All ethnic groups in the study area use a combination of different criteria to group enset landraces. We recorded three use categories, as defined by (25), in order of importance: Food (*kocho* yield and quality, *bulla* quality, *amicho* use), fiber (fiber quality) and medicinal/ritual value as described in Table 3. Of the 312 landraces: only 11 landraces having one use type, 56 landraces having two use types and a total of 245 landraces having more than two use types (Fig. 4). In addition, Fig. 5 shows the comparative result of the use categories according to the ethnic groups. Fair analysis between ethnic groups revealed that the highest value for food (*kocho* yield and quality) were (≥35 house hold/ethnic) observed in all ethnic groups.

Almost all of the landraces used for good *kocho* and *bulla* yield and quality have got a wider distribution and diversity (Table 5). The fiber uses showed higher values for all ethnic groups. Farmers also reported enset landraces having longer and/or stronger fibers, and higher fiber yield and quality (Table 6). Forty two landraces were identified by farmers for *amicho* use value (Table 7). In addition, some enset landraces are known by farmers to have medicinal value for both humans and animals. These landraces are poorly producing and to be maintained for special traditional or religious uses (Table 8). Almost all landraces in this category have got sweet *amicho* test therefore both categories share more than 50 % of the landraces. In addition to the above use value; farmers in each ethnic group use biotic and abiotic tolerance as a trait for diversity maintenance. Fifty and thirty three landraces were identified by farmers as tolerant to enset bacterial wilt and drought (Tables 9 & 10).

**Table 5:** Number of farmers who are growing the most abundant and widely distributed enset landraces per ethnic group

No.	Landrace name	Number of respondents (*N* = 40)									
		Da	Ge	Gu	Ha	Ke	Si	Sil	Wo	Total	ethnic group
1	*Ado*						34			34	1
2	*Agade*			38				38		76	2
3	*Ahero*							19		19	1
4	*Amiya*	15								15	1
5	*Argama*	17								17	1
6	*Arkeya*	21								21	1
7	*Astara*		31	21						52	2
8	*Badedet*	24		26				23		73	3
9	*Bazereye*			21						21	1
10	*Beneze*							21		21	1
11	*Bira*						16			16	1
12	*Birbo*						15			15	1
13	*Boser*			17						17	1
14	*Boza*	20								20	1
15	*Chacho*						15			15	1
16	*Dere*			19						19	1
17	*Dirbo*					16				16	1
18	*Desho*				28					28	1
19	*Enquafye*			18						18	1
20	*Etene*					18				18	1
21	*Ferezye*			23						23	1
22	*Genbo*				34	15		22		71	3
23	*Genna*						21			21	1
24	*Genticha*		37				39			76	2
25	*Guarye*							17		17	1
26	*Gulumo*						16			16	1
27	*Hiniba*							20		20	1
28	*Kinbat*							30		30	1
29	*Kiticho*						24			24	1
30	*Mazia*	28								28	1
31	*Merza*					16				16	1
32	*Midasho*						25			25	1
33	*Nefo*		23							23	1
34	*Qibnar*			17						17	1
35	*Seskela*				25	34				59	2
36	*Sheleqe/Shelequmia*					15			25	40	2
37	*Shirteye*			22				20		42	2
38	*Shododinia*	37								37	1
39	*Torore/Toracho*		20		19					39	2
40	*Tuzuma*								22	22	1
41	*Uwisho*						21			21	1
42	*Yaka*	22								22	1

*Da* Dawro, *Ge* Gedeo, *Gu* Gurage, *Ha* Hadiya, *Kem* Kembata-Tembaro, *Sid* Sidama, *Sil* Silte, *Wol* Wolayita

**Table 6:** List and distribution of Enset landraces reported by farmers for better fiber yield and quality

No.	Landrace name	Location	Frequency of respondents (*N* = 40)	No.	Landrace name	Location	Frequency of respondents (*N* = 40)
1	*Abatemerza*	Kembata-Tembaro	31	23	*Lemat*	Gurage	17
2	*Ayase*	Kembata-Tembaro	24	24	*Ankefuye*	Gurage	20
3	*Digmerza*	Kembata-Tembaro	28	25	*Enba*	Gurage	15
4	*Ferchase*	Kembata-Tembaro	23	26	*Yeshirakinke*	Gurage	32
5	*Zobira*	Kembata-Tembaro	19	27	*Gimbo*	Gurage	30
6	*Unjame*	Kembata-Tembaro	32	28	*Tikur Badadiet*	Gurage	24
7	*Sapara*	Kembata-Tembaro	30	29	*Teriye*	Gurage	25
8	*Gishira*	Kembata-Tembaro	32	30	*Bedade*	Gurage	30
9	*Disho*	Kembata-Tembaro	21	31	*Sabora*	Gurage	19
10	*Gishira*	Kembata-Tembaro	28	32	*Toracho*	Sidama	17
11	*Siskella*	Kembata-Tembaro	32	33	*Kiticho*	Sidama	14
12	*Gimbo*	Kembata-Tembaro	20	34	*Ado*	Sidama	26
13	*Shetadena*	Kembata-Tembaro	14	35	*Midasho*	Sidama	24
14	*Agade*	Kembata-Tembaro	18	36	*Gena*	Sidama	29
15	*Mazia*	Wolayita	24	37	*Wundiraro*	Sidama	16
16	*Bedade*	Wolayita	20	38	*Tsella*	Dawro	20
17	*Gefeteno*	Wolayita	26	39	*Kertia*	Dawro	18
18	*Halla*	Wolayita	32	40	*Yeka*	Dawro	22
19	*Godoria*	Wolayita	20	41	*Yesha Mazea*	Dawro	26
20	*Amaratye*	Gurage	22	42	*Bota Mazea*	Dawro	24
21	*Agade*	Gurage	24	43	*Mecha Boza*	Dawro	21
22	*Nechiwe*	Gurage	20

**Table 7:** List and distribution of Enset landraces reported by farmers for better *amicho* use quality

No.	Landrace name	Ethnic group	Frequency of respondents (*N* = 40)	No.	Landrace name	Ethnic group	Frequency of respondents (*N* = 40)
1	Sebera	Kembata-Tembaro	37	22	Tessa	Kembata-Tembaro	33
2	Switea	Wolaita	36	23	Fenqo	Gurage	30
3	Sirareia	Wolaita	33	24	Agade	Gurage	23
4	Bose	Kembata-Tembaro	29	25	Musula	Dawro	30
5	Leqaqa	Kembata-Tembaro	31	26	Bukuniya	Dawro	25
6	Neqaqa	Wolaita	29	27	Qibnar	Gurage	32
7	Bino	Kembata-Tembaro	26	28	Qoyina	Kembata-Tembaro	31
8	Shelequmia	Wolaita	33	29	Neqaqa	Dawro	33
9	Matiya	Dawro	30	30	Guariye	Kembata-Tembaro	34
10	Chohot	Gurage	35	31	Argema	Dawro	29
11	Diqa	Dawro	26	32	Arkiya	Dawro	32
12	Keteniya	GamoGoffa	30	33	Niffo	Gededo	33
13	Ashakit	Gurage	29	34	Addo	Sidama	29
14	Gena	Wolaita	32	35	Gedeme	Sidama	33
15	Switeia	Dawro	33	36	Qinware	Silte	32
16	Tuffa	Dawro	27	37	Agincho	Kembata-Tembaro	29
17	Zinka	Dawro	23	38	Tessa	Hadiya	26
18	Astara	Gurage	27	39	Darasicho	Sidama	29
19	Silqantia	Wolaiyta	29	40	Kiticho	Sidama	30
20	Sheleqe	Kembat-Tembaro	30	41	Disho	Kembata-Tembaro	28
21	Gazner	Gurage	33	42	Guarye	Silte	32

**Table 8:** List and distribution of enset landraces reported by farmers for their medicinal and ritual purposes

No.	Landrace name	Frequency of respondents	No.	Landrace name	Frequency of respondents
1	Addo	12	16	Garercho	15
2	Agade	15	17	Gesher	25
3	Agunited	13	18	Gulemo	17
4	Altecho	11	19	Qeqele	35
5	Arikiya	12	20	Keter	28
6	Askale	10	21	Lochinge	33
7	Astera	18	22	Merze	16
8	Badedet	20	23	Munderaro	19
9	Botate	19	24	Nerim	21
10	Chacho	20	25	Nifo	27
11	Cherkuwa	17	26	Qibnar	26
12	Chovet	22	27	Signore	28
13	Dem woured	31	28	Swetiya	30
14	Dere	29	29	Tenako	19
15	Guarye	28	30	Tesa	29

**Table 9:** *Xanthomonas wilt* tolerant cultivars reported/used by farmers in the eight surveyed ethnic group

No	Landrace name	Frequency of respondents (*N* = 40)	No	Landrace name	Frequency of respondents (*N* = 40)
1	*Addo*	24	26	*Gatecho*	26
2	*Agade*	20	27	*Gena*	32
3	*Ager amer*	13	28	*Ginbura*	21
4	*Agunta*	15	29	*Gishera*	24
5	*Ahiro*	19	30	*Gosala*	14
6	*Altecho*	12	31	*Kombat*	19
7	*Amiya*	17	32	*Kotecha*	20
8	*Argama*	20	33	*Kuruma*	26
9	*Ashekit*	21	34	*Kuruwa*	29
10	*Astara*	24	35	*Maziya*	32
11	*Badedit*	30	36	*Midasho*	28
12	*Banko*	19	37	*Nechwe*	25
13	*Baze*	20	38	*Nifo*	14
14	*Beker*	12	39	*Sesekela*	27
15	*Benezhe*	18	40	*Shodedine*	25
16	*Bera*	13	41	*Shasha*	18
17	*Berbo*	15	42	*Sheleqe*	20
18	*Degomerza*	18	43	*Shirteye*	13
19	*Dere*	22	44	*Tegeded*	15
20	*Dewarama*	18	45	*Tsela*	17
21	*Enba*	20	46	*Tuzmia*	19
22	*Enkufaye*	21	47	*Unjame*	22
23	*Etne*	24	48	*Wanadia*	20
24	*Gadami*	18	49	*Yesha maziya*	28
25	*Garado*	23	50	*Zegez*	21

**Table 10:** List and distribution of Enset landraces reported by farmers as drought tolerant

No.	Landrace name	Location	Frequency of respondents	No.	Landrace name	Location	Frequency of respondents
			(*N* = 40)				(*N* = 40)
1	*Toracho*	Sidama	24	18	*Kertia*	Dawro	19
2	*Genticho*	Sidama	28	19	*Shododina*	Dawro	23
3	*Nifo*	Sidama	19	20	*Yesha mazea*	Dawro	25
4	*Quarase*	Sidama	25	21	*Bota mazea*	Dawro	26
5	*Kiticho*	Sidama	27	22	*Attuma boza*	Dawro	22
6	*Ado*	Sidama	24	23	*Bonga arkia*	Dawro	17
7	*Midasho*	Sidama	29	24	*Ankefuye*	Gurage	24
8	*Gena*	Sidama	30	25	*Enba*	Gurage	20
9	*Gena*	Sidama	30	26	*Gimbo*	Gurage	29
10	*Wundiraro*	Sidama	27	27	*Tikur badadiet*	Gurage	27
11	*Ayase*	Kembata-Tembaro	23	28	*Teriye*	Gurage	23
12	*Sapara*	Kembata-Tembaro	26	29	*Bedade*	Gurage	30
13	*Gishira*	Kembata-Tembaro	22	30	*Sabara*	Gurage	25
14	*Unjame*	Kembata-Tembaro	24	31	*Beneze*	Gurage	20
15	*Disho*	Kembata-Tembaro	25	32	*Mazia*	Wolita	26
16	*Gimbo*	Kembata-Tembaro	28	33	*Halla*	Wolita	29
17	*Tsella*	Dawro	20				

### Indigenous knowledge on the management of enset diversity

People in the study area maintain their enset farm with considerable structured planting, diversity and flexibility that support production of this livelihood crop. They have managed to select landraces that adapt the local environment and that give multiple benefits. According to the information we obtained during individual interview, key informant and focus group discussion, management and maintenance of on-farm enset diversity is influenced by: (i) systematic propagation of the landraces, (ii) exchange of planting material (iii) selective pressure.

**Fig. 6:**
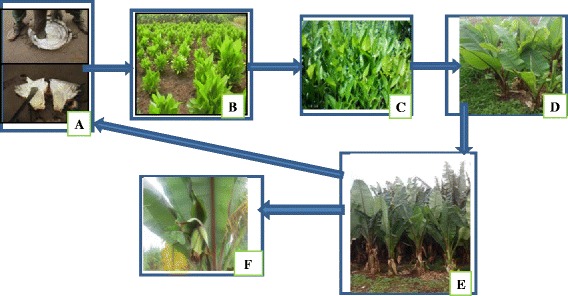
Systematic propagation of enset. **a** mother corm ready for burring; **b** suckers emerged from the mother corm; **c** 1st stage transplanting; **d** 2nd stage transplanting; **e** 3rd stage transplanting; **f** Matured enset ready for harvesting

(i) **Systematic propagation of the landraces**
  Systemic propagation of the landraces is practices used by all farmers in the study area to adjust and to maintain the landrace diversity. Almost all farmers in the study area use corms of 3 to 4 years old enset plants with some portion of the pseudostem to produce enset seedlings (Fig. 6 & Table 11).

**Table 11:** Type of planting materials used by Enset producing farmers

No.	Type of planting material	Frequency(*N* = 320)	Percent
1	Corm	238	73.7
2	Suckers	63	19.5
3	Corm & Suckers	10	3.1
4	Botanical seed	0	0
  Almost all respondents indicated that there are three to four growth stages or frequency of transplanting before harvesting (Table 12). The informants indicated that the propagation starts from the third stages of transplanting (Fig. 6e). Farmers traditionally practiced removal of the central shoot and removal of the apical dominance corms ready for burring (Fig. 6a). Hypothetical question posed in the interviews was what happen if you plant the corm without removal of the central part? The respondents indicated that the removal of the central area helps the propagated corm to produce more number of suckers (≥50 suckers /corm) for next season multiplication (Fig. 6b). The first sucker production stage stays 1 year after emergence from the buried corm (Fig. 6c). In the second stage, the produced multiple suckers from the buried mother corm detached and planted in rows with two to three suckers in a group, or in rows of single plants (Fig. 6d). A consecutive transplanting produces the third stage (Fig. 6e). Farmers’ indicated that the third stage is used as both the source of mother corm for sucker multiplication and harvested for consumption when there is less amount of food in the stock. At the end of the third stage, the suckers are transplanted a fourth time to the permanent field (Fig. 6f). The total time required from first planting to harvesting can be around 7–8 years. The propagation usually carried out in the dry season (November to early February). Farmers propagate a diverse landraces available in the farm. Some multipurpose landraces are propagated by the majority of households interviewed.

**Table 12:** Local names of the different enset transplanting stages

Location	1st stage	2nd stage	3rd stage	4th stage
Dawro	Halua	Bashashua	Gardwa	Wossa
Gedeo	Simma	Kassa	Satta	Daggicho
Gurage	Fonfo	Simma	Teket	Hiba
Hadiya	Dubo	Simma	Ero	Weasa
Kembata-Tembaro	Dubo	Simma	Ero	Ballessa
Sidama	Funta	Awulo	Qatalo	Daqicho
Silte	Bosho	Dafaro	Kiniba	Waise
Wolaita	Halua	Bashashiya	Gardwa	Wasa
(ii) **Exchange of planting material**
  Traditional planting material exchange system is an important source of diversity for majority of farmers. Out of the 320 farmers interviewed 249 farmers use corms from their own farms (Fig. 7). One fourth of the 320 farmers’ interviewees mentioned that they often hand out or sell corms/planting material to neighbors or fellow villagers. Neighbors, relatives, and market were the sources of planting material and exchange, gift, purchase and free distribution were the main bases of enset planting material flow. Planting material flow took place inside and outside the village.

**Fig. 7:**
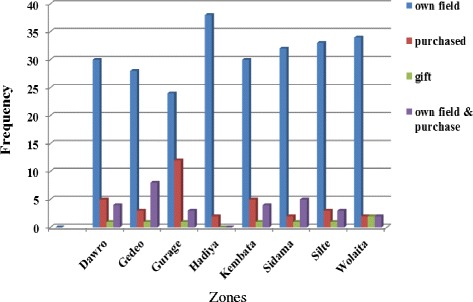
Source of planting material in the surveyed zones
(iii) **Selective pressure**
  Farmers continue to face many risks because of enset’s vulnerability to biotic and abiotic problems, and global climate change. Landraces which perform better under different biotic stress, and diverse agro-ecological conditions, and having multiple uses should be recommended to these subsistence farm households in order to sustain their livelihoods. Almost all informants stated that the population of enset has declined in recent times both in abundance and in distribution. The factors purportedly responsible for this decline were both agriculture and natural (disease and pest and drought) (Table 13)

**Table 13:** Most frequently reported enset production constraints in the study area

Major constraints in enset production	Reported by % of farmers?
Enset *Xanthomonas* Wilt	35.9
Enset root mealy bug	34.6
Leaf hopper	19.5
Mole rat	24.7
Porcupine	52.2
Swine	12.4
Corm rot	52.8
Drought	8.9
  Almost all farmers’ reported that Enset *Xanthomonas* wilt (EXW) had the greatest impact on enset production. Nearly 36 % of farmers reported the existence of EXW in their fields (Table 13). Each respondent was able to name a significant number of vernacular names though not all landraces are planted and maintained in his or her backyard. Prior to the arrival of EXW, farmers in the region would have selected enset landraces for a number of traits. However; this disease causes complete death of the plant within weeks after the first symptoms and it has completely wiped out enset in some areas. The disease has forced farmers to abandon enset production resulting in critical food shortage in the densely populated areas of southern Ethiopia. It is now recognized as a national problem, having increased in severity.

## Discussion

### Strategic importance of enset

Enset is well-established, sustainable, and environmentally resilient farming system that contributes to food security of farmers and, in particular it serves as food security crop in densely populated areas. Enset needs to be present in farmers’ pits throughout the year. Enset is the most important crop in the region. According to 2011 CSA [16] report 3,020,143 km^2^ of land is covered by enset crop and about 6.9 million quintals of enset yields were produced in 2010/11 production season.

All farmers are using the landraces developed by the community [21]. These landraces have been grown on-farm thousands of years. These enset-growing traditions still continue in the current generation. Enset represents an important cultural plant in the region. This appreciation is consistent with previous studies on the crop [4, 6, 13, 14, 22, 23]. Such cultural importance is reflected in the multiple uses of enset in the traditional ecological knowledge about the crop, its biological attributes, morphological and quality variation, including size, yield and other use value quality recognized by local people among the different ethnic groups.

### Indigenous knowledge in naming and classification

Farmers’ rich knowledge that is accumulated on the crop over many years has played a significant role in naming, characterization and maintenance of the existing genetic diversity. Enset producing farmers have their own folk naming and classification system to distinguish one landrace from the other. Sometimes it is difficult to understand and reclassify, even while watching them to characterize. The classification of enset landraces has been accommodated by phenotypic differences, unique traits and specific uses of landraces. As pointed out by [4, 13, 14], these are common characteristics of folk classification systems in enset. Folk nomenclature is an integral part of the variety management in enset farming systems [24, 25]. In view of this, the multitude of names in various folk taxonomic levels indicated the occurrence of on farm genetic diversity at infra-specific level. As indicated by [13], landrace names given by farmers’ have been used as farmers’ diversity unit for estimating unit for the extent and distribution of enset diversity as well as *ex situ* collection. This is also in agreement where folk taxonomy is used to highlight the amount of genetic diversity [18, 26, 27]. In this study, over 300 landrace names (Table 14) have been identified which indicated the level of on farm genetic diversity. The meaning of the names of most landraces is not known. It is difficult to know unless the people who named it or the place of origin are traced back. It has been repeatedly reported that unexplained meanings of folk names were common in other ethnic groups [13]. A similar pattern was observed in other crops like sorghum and rice [28, 29]. Enset landraces were commonly exchanged and distributed according to the folk names. The finding of this study (Table 5) and other similar studies [13, 14, 21] depict identically named landraces were also reported in more than one ethno-linguistic communities. Folk classification can help in identifying the comparative value of landraces (for example Tables 6, 7 & 8) for proper characterization and pre-breeding activities. A similar study on sorghum in Ethiopia [28] and rice in Nepal [29] has shown that name of the varieties indirectly related showed the functional value for the variety.

**Table 14:** List of named clones in the eight ethnic groups, Diversity of the clones and richness of the Zones

No	Name of the clone	Silte	Gur	Kem	Had	Wol	Daw	Ged	Sid	TOT	Zones	1-D	Evenness
		Frequency											
1	agede	38	38		5		2			83	4		
2	ager amer	11								11	1		
3	ahero	19	6	1						26	3		
4	anzene	2								2	1		
5	asheket	3	1							4	2		
6	ashure	2		1						3	2		
7	astera	6	21	2	1			31		61	5		
8	aywepe	8								8	1		
9	badedit	23	26	1	1		24			75	5		
10	bamlia	4		2						6	2		
11	bazereye	1	21							22	2		
12	beneze	21	3	1	6					31	4		
13	boseda	1								1	1		
14	boser	10	17							27	2		
15	chigezh	1								1	1		
16	dem werer	6								6	1		
17	dere	10	19	3						32	3		
18	dereketa	2		2						4	2		
19	Dirbo/Dirbwa	2	2	16	4					24	4		
20	enkufaye	7	18							25	2		
21	Etnete	1		18	1					20	3		
22	eyase	1		2						3	2		
23	fechecho	1		2						3	2		
24	ferezeye	6	23							29	2		
25	gafet	4								4	1		
26	gareye	17	12	3						32	3		
27	genbo	22	10	15	34					81	4		
28	geradiye	1								1	1		
29	ginbura	1								1	1		
30	ginjina	1		2	1					4	3		
31	gomboter	2		1						3	2		
32	guder	3								3	1		
33	hinib	20		1						21	2		
34	kaker ginbo	2								2	1		
35	kaset	2		1	6					9	3		
36	keter	1								1	1		
37	kibnar	11		1						12	2		
38	kinbat	30	6							36	2		
39	kogogot	1								1	1		
40	kombeter	1								1	1		
41	lemat	1	8							9	2		
42	meriye	2		6	8					16	3		
43	mintigre	3								3	1		
44	moche	4	1	4	9				1	19	5		
45	nechewo	3	15	2						20	3		
46	sebera	9		2						11	2		
47	sesekila	4		34	25					63	3		
48	setner	2		3						5	2		
49	shesha shirteye	3								3	1		
50	shirteye	20	22	1			1			44	4		
51	showrat	9			1					10	2		
52	sino	6								6	1		
53	sorat yebadedit	3	1							4	2		
54	tegeded	11	7	2						20	3		
55	tereye	1	4							5	2		
56	torore/Toracho	3	1	6	19	2	1	20	1	53	8		
57	uzkurz	1		3	8					12	3		
58	Wahe,a	1		4	1					6	3		
59	woshamada	7		3						10	2		
60	welegele	1								1	1		
61	wunado	3	1		6					10	3		
62	yedebir	3								3	1		
63	yesherafere	8	9							17	2		
64	yezer badedit	3	4	1						8	3		
65	zagez	1								1	1		
66	zebre	1								1	1		
67	zeget	2	1							3	2		
68	zelebedadit	5	2	1						8	3		
69	zigiz	1								1	1		
70	amerat		4							4	1		
71	anash		3							3	1		
72	argama		1	4	1	1	17			24	5		
73	art		1							1	1		
74	aseso ert		1							1	1		
75	azina		2							2	1		
76	baritsya		1							1	1		
77	botena		1							1	1		
78	boza		2				20			22	2		
79	bukuniya		1				7			8	2		
80	chehoyet		4							4	1		
81	emreye		7	1						8	2		
82	enba		2							2	1		
83	gasa		1							1	1		
84	genbene bazereye		1							1	1		
85	genna		1			6	4		21	32	4		
86	gezit		2							2	1		
87	kanchuwe		3		1					4	2		
88	katania		2				3			5	2		
89	Kekle		2	5						7	2		
90	kuanchewe		1							1	1		
91	kushkusheye		2							2	1		
92	natsam		1							1	1		
93	nech bazer		1							1	1		
94	neriye		2							2	1		
95	qey b azer		3	2						5	2		
96	qibnar		17							17	1		
97	serat		5							5	1		
98	sheme agaye		1							1	1		
99	tederader		5							5	1		
100	woret		1							1	1		
101	yeilma		1							1	1		
102	yekela enset		1							1	1		
103	yergeye		1							1	1		
104	zegurt		1							1	1		
105	abet merze			5						5	1		
106	ambo			1						1	1		
107	aniya			1						1	1		
108	banko			2						2	1		
109	cherkuwa			1	1					2	2		
110	dego			8	2					10	2		
111	desho			6	28					34	2		
112	diqaa			1						1	1		
113	farachase			2						2	1		
114	gesher			15	10					25	2		
115	goderete/Godere			1		1				2	2		
116	gonmora			1						1	1		
117	haeala			6		8				14	2		
118	keberbeye			1						1	1		
119	koyena			2	6					8	2		
120	lekaka			15	1					16	2		
121	menduleka			1						1	1		
122	mereze			16	7					23	2		
123	mesmes/Mesmesiya			2	10	1				13	3		
124	sheleqe			15	8					23	2		
125	shesha shirteye			2						2	1		
126	sorpe			12						12	1		
127	tebere			2						2	1		
128	tesa			6	5					11	2		
129	udole			1						1	1		
130	unjamo			16	9					25	2		
131	wacheso			2						2	1		
132	walema			1						1	1		
133	wolanche			5	2					7	2		
134	Bekuch				3					3	1		
135	Bose				3					3	1		
136	Ezgera				2					2	1		
137	Fuga				1					1	1		
138	Gozod				2					2	1		
139	Haywena				10					10	1		
140	hekecha				1					1	1		
141	Henuwa				5					5	1		
142	Kekir				1					1	1		
143	Korin				2					2	1		
144	Lokenda				3					3	1		
145	separa				10					10	1		
146	Shate				5					5	1		
147	Shodedina				2					2	1		
148	Shumbiratie				1					1	1		
149	Sinere				6					6	1		
150	Sinkute				1					1	1		
151	Sowandiya				1					1	1		
152	Ti'ona				1					1	1		
153	Zobira				4					4	1		
154	ankogena					2	1			3	2		
155	alagena					9				9	1		
156	anekuwa					4				4	1		
157	arekiya					6	21			27	2		
158	atane					1				1	1		
159	botiya					2				2	1		
160	chemeya					3				3	1		
161	checheya					1				1	1		
162	Dinka					1				1	1		
163	gefetanuwa					12				12	1		
164	Lenbo					5				5	1		
165	lochanegeya					2	7			9	2		
166	Mazia					4	28			32	2		
167	naqaqa					11				11	1		
168	qabarecho					4				4	1		
169	qabariya					15				15	1		
170	qucha					1				1	1		
171	shala qomiya					25	1			26	2		
172	sutiya					1	1			2	2		
173	tuzuma					22	5			27	2		
174	wanaqbariya					2				2	1		
175	wanadeya					10				10	1		
176	adinona						2			2	1		
177	adnar						1			1	1		
178	agina						7	4	3	14	3		
179	agunsa areziya						1			1	1		
180	alodnita						1			1	1		
181	amiya						15			0	1		
182	amraga						1			1	1		
183	anko maziya						6			6	1		
184	ante argal						1			1	1		
185	areteya						1			1	1		
186	bakiya						1			1	1		
187	bala arkiya						2			2	1		
188	bale geziya						1			1	1		
189	bale maziya						1			1	1		
190	bale shedodeniya						2			2	1		
191	barjia						1			1	1		
192	betaniya						1			1	1		
193	betsena						2			2	1		
194	banga						1			1	1		
195	bosena						12			12	1		
196	bota maziya						5			5	1		
197	botindira						2			2	1		
198	deka						1			1	1		
199	deka arikiya						2			2	1		
200	digaa						1			1	1		
201	ealoria						2			2	1		
202	erantia						2			2	1		
203	gadeye						1			1	1		
204	gamaria						2			2	1		
205	giea						1			1	1		
206	hal maziya						7			7	1		
207	hoindia						4			4	1		
208	kareta mati						1			1	1		
209	kartiya						8			8	1		
210	kekefeya						4			4	1		
211	keruma						9			9	1		
212	koziya						1			1	1		
213	kuruwa						12			12	1		
214	macha shededin						1			1	1		
215	manjo maziya						1			1	1		
216	mataka						7			7	1		
217	mushwa						1			1	1		
218	samra						3			3	1		
219	sanka						6			6	1		
220	shedodeniya						37			37	1		
221	shemoya						3			3	1		
222	shemta						1			1	1		
223	shesha						2			2	1		
224	shuchfin						2			2	1		
225	sirara						4			4	1		
226	tsela						13			13	1		
227	woaya						2			2	1		
228	yaka						22			22	1		
229	yapa						9			9	1		
230	yerga						1			1	1		
231	yesha						3			3	1		
232	yesha maziya						9			9	1		
233	yiliga						6			6	1		
234	zira maziya						3			3	1		
235	Denbola							8		8	1		
236	deneka							2		2	1		
237	Dimoye							8		8	1		
238	filil							2		2	1		
239	fokonie							2		2	1		
240	Foneqe							2		2	1		
241	Galasho							1		1	1		
242	ganetecho							37	39	76	2		
243	Gatara							2		2	1		
244	Gosalo							4	10	14	2		
245	haramo							7		7	1		
246	haranjo							1		1	1		
247	Helila							1		1	1		
248	kake							1		1	1		
249	Mundame							3		3	1		
250	nefo							23	4	27	2		
251	Qarasie							15		15	1		
252	qelitate							1		1	1		
253	qeralicho							1		1	1		
254	qorqor							2		2	1		
255	shasha							2		2	1		
256	Shegna							2		2	1		
257	toramy							6		6	1		
258	adem ado								2	2	1		
259	addo								34	34	1		
260	alom a								1	1	1		
261	altecho								9	9	1		
262	arsho								2	2	1		
263	askale								14	14	1		
264	aydira								1	1	1		
265	batota								3	3	1		
266	berberachu								1	1	1		
267	bericho								1	1	1		
268	bero gantecha								1	1	1		
269	bewot ado								2	2	1		
270	bira								16	16	1		
271	birbo								15	15	1		
272	birdere								1	1	1		
273	bonjo								6	6	1		
274	borganticha								6	6	1		
275	bufere								4	4	1		
276	bulo								6	6	1		
277	chacho								15	15	1		
278	damala								2	2	1		
279	derese ado								3	3	1		
280	dersem								1	1	1		
281	dersete								11	11	1		
282	dewane								1	1	1		
283	deweramo								6	6	1		
284	enboma								3	3	1		
285	gabewo								3	3	1		
286	gademe								12	12	1		
287	gamachala								2	2	1		
288	garbo								1	1	1		
289	goloma								1	1	1		
290	gulumo								16	16	1		
291	haho								3	3	1		
292	hamsesa								1	1	1		
293	hawe								1	1	1		
294	hekece								1	1	1		
295	kanda								1	1	1		
296	keshe								6	6	1		
297	kiticho								24	24	1		
298	kule								10	10	1		
299	lemecho								4	4	1		
300	mada								4	4	1		
301	mendenar								8	8	1		
302	midasho								25	25	1		
303	monofila								1	1	1		
304	nech enset								1	1	1		
305	resecho								1	1	1		
306	sercho								1	1	1		
307	serero								2	2	1		
308	sidera								1	1	1		
309	uwisho								21	21	1		
310	wankore								2	2	1		
311	washa								1	1	1		
312	worm kalo								1	1	1		
Richness of zones		69	63	66	51	28	75	26	62				
Number of rare clones		21	26	15	20	15	58	20	55				

*Da* Dawro, *Ge* Gedeo, *Gu* Gurage, *Ha* Hadiya, *Kem* Kembata-Tembaro, *Sid* Sidama, *Sil* Silte, *Wol* Wolayita

Commonly, knowing folk names and classification may distinguish varieties that are actually genetically very closes. Farmer’s in one household generally knows which households certainly have named varieties and their particular agronomic and use value related characteristics. Knowing folk taxonomy also helps in developing planting material distribution, flow channels, and regional landrace map. Thus, even if landrace names and classification are a necessary basis, they are not sufficient to describe genetic diversity. Integrative indicators have been designed e.g., complementing the naming and folk classification with parameters of genetic diversity. Our data thus needs to be complemented by phenotypic and genotypic information which helps to avoid redundancies and optimizing the efficient conservation and sustainable use of the crop.

### Level of on-farm richness, diversity and pattern of use

Enset farming systems are rich in landraces diversity. In the study area we recorded a relatively high landraces (312) richness of enset. For instant, in previous studies, comparable results were reported by [21], who described 218 different enset landraces from seven ethnic groups. One hundred eleven enset landraces were also reported from nine growing areas of Ethiopia [7], while [13] described 67 enset landraces from Wolaita zone of the southern region. The number of enset landraces in this study is far higher than what was reported by previous studies which were conducted in zones with similar climatic and altitudinal factors. For instance, [21] reported the presence of 41 landraces in Dawro, which is far below the number of enset landraces reported in the present study. During discussion with the farmers it has been observed that, there were more than 100 enset landraces grown in each locality a few years back, however, farmers reported that most of the landraces were lost due to EXW. Tesfaye [24] also found out that in Sidama zone farmers reported names of 20 enset landraces which were not encountered in any of the farms that were visited. Some enset landraces might have been totally lost from farmers’ fields.

Enset is a multipurpose crop which is utilized for different use values. Based on their use value and folk classification large differences were evident between landrace abundance and distribution in the region. Some landraces, particularly those having merits of better *kocho* yield and quality have got a wider distribution within and between ethnic groups/zones. For example, the enset landraces ‘*Shododenia*’ and ‘*Addo’* were encountered on respectively 37 and 34 (92.5 and 85 %) farms visited in Dawro and Sidama, but were not found in any other surveyed zones. Some landraces had a very high local abundance at one or two locations and were absent from the rest. For example *Shodedenia* was encountered on 100 % of the farms visited in Dawro. It was encountered on all the 40 (100 %) farms visited in Dawro. Likewise, [24] reported a small number of landraces (for instant *Genticha*) playing a dominant role in Sidama zone. Our study revealed that the highest use values of the landraces were found in the region which also corresponds to where the landraces had the highest abundance in the farming system. This suggests a positive relationship between plant abundance and use. These findings corroborate the “apparency hypothesis” which describes dominant, large and more abundant plant species as having the highest use values.

Enset bacterial wilt, caused by *Xanthomonas campestris* pv. *musacearum*, is the most important biotic constraint to enset cultivation [6]. In order to alleviate this biotic stress farmers integrate EXW tolerant landraces in their farms. The *kocho* yield of these disease tolerant landraces is however below average [26, 27]. Moreover, some enset landraces are known by farmers to have medicinal value for both humans and animals. These landraces are most often poor yielding and are only maintained for special traditional or religious purposes/uses. Those landraces are reported to heal bone fractures, are used for treating diarrhea and during child delivery i.e., assisting the discharge of the placenta. Most reports of medicinal and ritual uses of enset indicate that farmers’ intentionally maintain the landraces together with other landraces. For example, [27] described 14 enset landraces based on their medicinal and ritual use value. Likewise, [26] reported a number of different enset landraces to have medicinal and religious (ritual) significance for preventive treatment, healing and other therapeutic purposes and as protection against evil spirits. Farmers also categorize enset landraces as male or female based on different characteristics [21, 30, 31]. However, the designation of landraces as ‘male’ or ‘female’ is not linked to their reproductive biology. According to farmers, the male enset landraces are drought tolerant. This designation is very important for maintaining landraces for *amicho* use value. Female landraces are described by farmers as less vigorous, susceptible to disease, having a higher *kocho* quality and producing edible and tasty *amicho* [31]. In addition, they are early maturing and have poor fiber strength. Surprisingly, few landraces have more than one use value. For example, the landraces ‘*Astara*’ and ‘*Addo*’ are known for their *kocho* yield and fiber quality. Similarly, in the Kembata area the landrace ‘*Siskela*’ is maintained by farmers for its high fiber yield and quality in addition to its high *kocho* yield. Studies by [14, 25] revealed that in most ethnical groups farmers maintain a single landrace for multiple uses. In some cases, poorly producing landraces continue to be maintained for special traditional (e.g., medicinal value) or religious uses. Farmers often maintain low yielding landraces that have medicinal values [25]. Similar observations have been made in banana-based communities in Uganda [32] or in rise systems in Asia [33].

Knowledge of the local usage of enset resources is essential for the elaboration of conservation strategies. This is the first time that the use values according to various ethnic groups in the study area have been evaluated in detail for enset. Overall, we found less diverse ethnic variation in knowledge and use values of enset, as has been found for difference within the same ethnic group [13, 14]. In general, this study and the previous studies have shown that different ethnic groups in the enset farming system demonstrated the existence of considerable amount of indigenous ethnobotany knowledge. High landrace diversity in a region may indicate extended periods of enset cultivation and a more subsistence form of production.

### Indigenous knowledge on the management of enset diversity

In the region, farmers’ manage local enset landraces within traditional production and processing systems oriented towards meeting household subsistence needs. Both women and men as producers, selectors, processors and marketers of enset are traditionally the custodians of *in situ* conservation. Farmers generally choose planting material from their existing mats. Farmers plant their enset landraces mixed on their fields, usually two or ten, but sometimes up to 20 landraces in one plot. It is traditional to use a corm and sucker as planting material and use of different transplanting stages in enset producing farmers. It was found that many households could propagate enset landraces in at least two ways and this flexibility of propagation might also reflect a relative preference for growing in a large area. A similar observation was also reported in other enset growing areas [13, 30, 31]. However it is yet to be identified whether such variations in propagation have some implications on maintenance of diversity *in situ*. Farmers observe and select the landraces based on their planting intentions for the coming year than the proportion to the quantity they have. This scenario has been maintained by the systematic propagation of 3–4 years old enset landraces. Other study [13] revealed that regular propagation and harvesting restrain; organized assemblage and arrangement of landraces in the home gardens and landrace composition regulation in the home gardens have been the major factor for indigenous management and maintenance of enset landraces on-farm. The rich selection experience on indigenous crop such as enset is also applied to other crops like sorghum [24].

The number of landraces grown at a given locality, their genetic similarity and the areas they occupy over time and space are influenced by planting material source, exchange and supply. Most planting material exchange is local, though a small proportion extends beyond the local group of villages reflecting relationships among neighbors and kin in most cases. All landraces used in the region are local farmer-named varieties. Among the surveyed farms, most farmers produce their own planting material. In addition farmers in the region have fixed systems to ensure the sustenance of planting material supply for each season. Farmers in cereal based farming system have well-established systems to ensure self-sustaining seed supply system and they often operate the exchange of planting material in the local market [34]. In general, on-farm conservation enhances continued source and supply of genetic material and continued diversity-based agriculture as compared to monoculture by ensuring intraspecific and interspecific diversity of crops. Farmers themselves perceived an advantage in continuing to grow diverse traditional crops and their participation in conservation of a traditional seed system proved to be self-sustaining.

Similarly farmers in the region quite frequently practices grow their landraces in mixture to stabilize their crop production, especially under adverse growing condition. Farmers may retain their preferred landraces over many years, often claiming they received no external inputs of seed/planting material. Plant diseases can also reduce the level of biodiversity or limit the variety of plants grown in an area. It have been observed that, the genetic base has been vulnerable to a range of very damaging biotic and abiotic stresses such as Enset *Xanthomonas* wilt (EXW), enset root mealy bug, leaf hopper, mole rat, Porcupine, wild pigs, corm rot, and drought. It is the EXW which has had the greatest impact on enset production. In Hadiya zone Lemu wereda 30 % of enset crop affected by EXW [35]. Therefore, farmers are forced to develop their copping strategies. Almost all surveyed farmers in the region practice cropping and dietary patterns change and grow more number of disease resistant plants as a strategy for the management of the disease. For instance, [36] indicated genetic diversity can be seen as a defense against problems caused by genetic vulnerability. To reduce the likelihood of spread, establishment and growth of EXW in enset crops, a systematic operational approach to the management of EXW should be adopted. This should include giving training to farmers on appropriate production practices, using healthy suckers and planting in clean soils. Future efforts surely need to focus on developing core collections representative of the widest possible genetic diversity for enset improvement and using this to strengthen *in situ* or on farm conservation.

## Conclusion

The information collected in the region and presented here shows that a certain wealth and diversity of knowledge regarding traditional naming, uses of plants and diversity management as a part of the cultural heritage of the community. Farmers’ have been growing enset for many years. The farmers’ knowledge and enset have been coevolving together. This has resulted in the prevalence of rich indigenous knowledge of the farmers. Any attempt to improve the crop needs to take in to account the farmers knowledge and experience.

Folk naming and classification are not consistent across all ethnic groups. The inconsistency is highly related with the ethnolinguistic variation in the region. Integrated folk-formal classification and characterization will be imperative for management and utilization of on farm genetic resources.

Our study confirms that the landrace diversity and distribution makes it possible to gain a general picture of the uses made of such crop on a macro-scale. A principal conclusion from the present study is that the biggest uses of landraces, in terms of the number of citations in the literatures, are for *kocho*, *bulla*, *amicho*, fiber and medicine. Certain traditional practices (for example spiritual or rituals) also lead farmers to maintain small quantities of uncommon landraces that may not produce well. This scenario points to the importance of use value based and other criteria similarity and differences for landrace diversity maintenance and management. Hence, formal enset improvement program needs to positioned in to multipurpose enset variety development scheme and include farmers and their knowledge in the research-extension continuum.

Landrace diversity in the region is affected by a number of factors. EXW is the main factor limiting enset richness and diversity. Any attempt to improve enset has to give emphasis on enhancement of farmers’ varieties and a systematic operational approach to the management of EXW.

It can be concluded that the existing farmers’ knowledge on naming, classification and diversity should be complemented with maintenance of the creative dynamics of traditional knowledge and transmission of the knowledge are crucial for constructing sustainable management.

## Abbreviations

EXW: Enset *Xanthomonas* wilt
SNNPRS: Southern Nations, Nationalities and Peoples’ Regional State
